# Nitrogen addition delays the emergence of an aridity-induced threshold for plant biomass

**DOI:** 10.1093/nsr/nwad242

**Published:** 2023-09-12

**Authors:** Hailing Li, César Terrer, Miguel Berdugo, Fernando T Maestre, Zaichun Zhu, Josep Peñuelas, Kailiang Yu, Lin Luo, Jie-Yu Gong, Jian-Sheng Ye

**Affiliations:** State Key Laboratory of Herbage Improvement and Grassland Agro-Ecosystems, College of Ecology, Lanzhou University, Lanzhou730000, China; Department of Civil and Environmental Engineering, Massachusetts Institute of Technology, Cambridge, MA 02139, USA; Instituto Multidisciplinar para el Estudio del Medio “Ramón Margalef,” Universidad de Alicante, Alicante 03690, Spain; Institut de Biologia Evolutiva (CSIC-UPF), Barcelona08003, Spain; Instituto Multidisciplinar para el Estudio del Medio “Ramón Margalef,” Universidad de Alicante, Alicante 03690, Spain; Departamento de Ecología, Universidad de Alicante, Alicante 03690, Spain; School of Urban Planning and Design, Peking University Shenzhen Graduate School, Peking University, Shenzhen518055, China; CSIC, Global Ecology Unit CREAF-CSIC-UAB, Barcelona 08193, Spain; CREAF, Cerdanyola del Vallès, Barcelona 08193, Spain; High Meadows Environmental Institute, Princeton University, Princeton, NJ 08544, USA; State Key Laboratory of Herbage Improvement and Grassland Agro-Ecosystems, College of Ecology, Lanzhou University, Lanzhou730000, China; State Key Laboratory of Herbage Improvement and Grassland Agro-Ecosystems, College of Ecology, Lanzhou University, Lanzhou730000, China; State Key Laboratory of Herbage Improvement and Grassland Agro-Ecosystems, College of Ecology, Lanzhou University, Lanzhou730000, China

**Keywords:** aboveground biomass, root:shoot ratios, elevated CO_2_, nitrogen fertilization, ecosystem threshold

## Abstract

Crossing certain aridity thresholds in global drylands can lead to abrupt decays of ecosystem attributes such as plant productivity, potentially causing land degradation and desertification. It is largely unknown, however, whether these thresholds can be altered by other key global change drivers known to affect the water-use efficiency and productivity of vegetation, such as elevated CO_2_ and nitrogen (N). Using >5000 empirical measurements of plant biomass, we showed that crossing an aridity (1–precipitation/potential evapotranspiration) threshold of ∼0.50, which marks the transition from dry sub-humid to semi-arid climates, led to abrupt declines in aboveground biomass (AGB) and progressive increases in root:shoot ratios, thus importantly affecting carbon stocks and their distribution. N addition significantly increased AGB and delayed the emergence of its aridity threshold from 0.49 to 0.55 (*P* < 0.05). By coupling remote sensing estimates of leaf area index with simulations from multiple models, we found that CO_2_ enrichment did not alter the observed aridity threshold. By 2100, and under the RCP 8.5 scenario, we forecast a 0.3% net increase in the global land area exceeding the aridity threshold detected under a scenario that includes N deposition, in comparison to a 2.9% net increase if the N effect is not considered. Our study thus indicates that N addition could mitigate to a great extent the negative impact of increasing aridity on plant biomass in drylands. These findings are critical for improving forecasts of abrupt vegetation changes in response to ongoing global environmental change.

## Teaser

Nitrogen addition delays the emergence of an aridity-induced threshold beyond which plant aboveground biomass abruptly decreases, thereby effectively mitigating the negative effects of projected aridification on plant biomass stocks in water-limited environments.

## INTRODUCTION

Aridity is a key climatic feature that measures the balance between the amount of water received via precipitation and that taken up by the atmosphere (potential evapotranspiration) [1–3]. Recent studies have shown that the crossing of certain aridity thresholds can lead to abrupt decays in ecosystem attributes such as plant productivity and increased plant mortality, thus constraining carbon stocks across global drylands [4–8]. Moreover, irreversible changes might occur once these thresholds are crossed, potentially causing widespread ecosystem degradation [4,9,10].

Using remotely sensed vegetation data from global drylands, Berdugo *et al.* (2020) [4] found abrupt decays in plant productivity and cover at aridity thresholds of 0.54 and 0.79, respectively. While this finding highlights the abrupt response of key vegetation attributes to the aridity gradient observed across global drylands, three key knowledge gaps remain to be addressed. First, it is unknown whether similar thresholds exist for field measured plant biomass, which is a key carbon sink and plays an essential role in protecting soil against erosion [11–16]. Second, it is also unknown how belowground biomass and the root:shoot ratio (the biomass ratio between roots and shoots) respond to increases in aridity across global drylands. Last, it is also unclear whether these aridity thresholds can be altered by the ongoing atmospheric enrichment of CO_2_ and nitrogen, two key drivers of global environmental change that largely affect plant photosynthesis and water use efficiency, and thus biomass and productivity [17–20]. Because of these knowledge gaps, our projections of how plant biomass may respond to climate change remain highly uncertain [21,22].

Elevated CO_2_ can reduce plant stomatal conductance and transpiration rates, and thus increase water use efficiency [17,23–25]. This water-saving process conserves soil water for greater carbon uptake and enhances plant responses to water stress [3,26]. Indeed, elevated CO_2_ has been identified as the major driver of the vegetation greening observed in many drylands worldwide in recent decades [3,27]. Nitrogen (N) is also a major limiting factor for plant growth in drylands [28]. Plant N supply and photosynthesis are closely linked, as demonstrated by the universal increase in photosynthesis with increasing leaf N content [29–31]. Therefore, anthropogenic increase in N via fossil-fuel combustion and fertilizer application frequently improves plant water use efficiency by stimulating photosynthesis [32–35]. The higher water use efficiency under higher N would conserve soil water and alleviate water stress [3,26]. However, both N and CO_2_ enrichments have been found to increase aboveground biomass, which may intensify soil water consumption and the negative effects of drought stress on plant biomass [3,36–38]. Therefore, it is unclear whether or not—and how—these two factors alter observed aridity-induced thresholds in drylands.

Here we combined >5000 empirical measurements of plant biomass from the literature and a multi-model simulation of the leaf area index (LAI, a proxy of foliage biomass with a high degree of certainty in model predictions) [27,39], to test the hypothesis that CO_2_ and N enrichments may alleviate plant water stress, and thus mitigate or delay the abrupt decay of plant biomass driven by aridity. To test this hypothesis, we investigated changing patterns of plant biomass along aridity gradients across areas with water deficit worldwide and evaluated the impacts of CO_2_ and N enrichments on the presence of aridity-induced thresholds. We compiled above- and belowground biomass (AGB and BGB, respectively), root:shoot ratio and AGB data measured under CO_2_ and N enrichments. Due to the limited availability of AGB measurements under CO_2_ enrichment in drylands [18], we also evaluated the impact of CO_2_ enrichment on the aridity threshold by coupling remote sensing LAI and model-simulated CO_2_ effects on LAI (an ensemble of 12 process-based dynamic vegetation models and a machine learning random forest model).

## RESULTS

### Nonlinear responses of plant biomass to aridity

**Figure 1.**
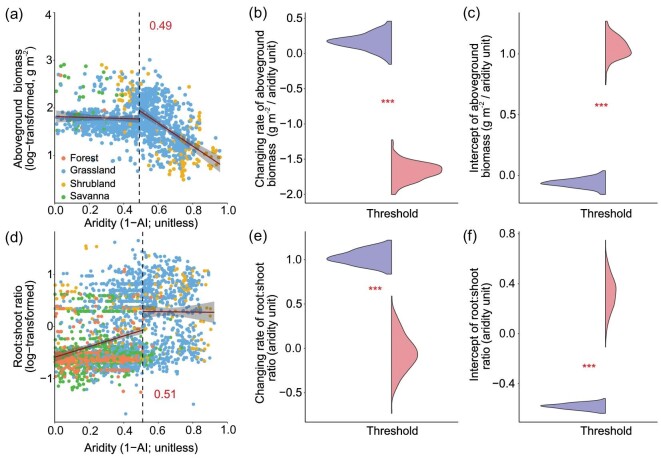
Nonlinear responses of plant biomass to aridity. Aridity thresholds for measured (a) aboveground biomass (*n* = 1353) and (d) root-shoot ratio (*n* = 3093). Solid red lines represent the linear fits on both sides of each threshold. The red numbers and vertical dashed lines represent the identified aridity thresholds. Plant biomass data were log-transformed to conform to normality. The violin diagrams in panels b and e show bootstrapped slopes of the predicted fitted trend at the threshold of the two regressions existing at each side of the threshold (purple before the threshold, red after the threshold). The violin diagrams in panels c and f show bootstrapped intercepts. The asterisks represent a significant difference before and after the aridity threshold at *P* < 0.001 using the Mann–Whitney U test.

Field measurements of plant above- and belowground biomass and the root:shoot ratio showed nonlinear responses to aridity, with thresholds occurring at an aridity value of ∼0.50 (Fig. 1; S3). This aridity value marks the transition from dry sub-humid to semi-arid climates. At low levels of aridity, AGB slightly decreased with increasing aridity. Once a 0.49 aridity threshold was crossed, however, AGB decayed abruptly (Fig. 1a–c). Belowground biomass exhibited a nonlinear response to aridity, with thresholds occurring at an aridity value of 0.52 (Figure S2a–c). Plants tend to allocate more biomass to roots than to shoots as aridity increases (Fig. 1d). This allocation was more obvious beyond the aridity threshold identified, as the root:shoot ratio step increased after aridity exceeding a 0.51 threshold (Fig. 1d–f). When we accounted for the influence of vegetation type and soil total nitrogen, the observed aridity threshold remained consistent (Figure S3a–i).

### Impacts of nitrogen and CO_2_ enrichment on plant aboveground biomass and the aridity threshold

**Figure 2.**
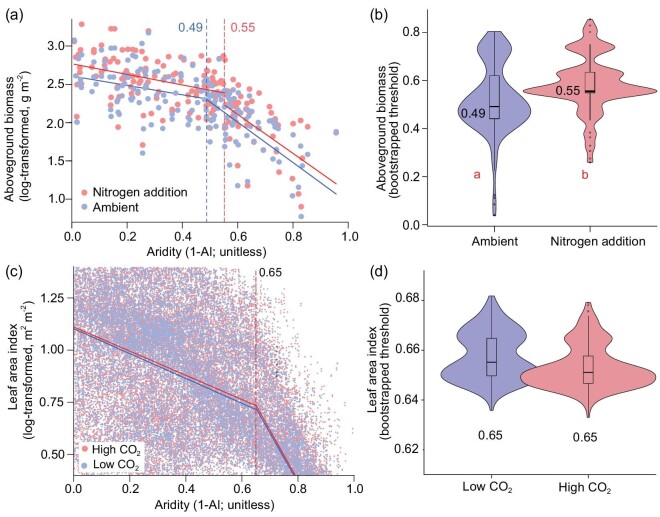
Impacts of nitrogen and CO_2_ enrichment on plant aboveground biomass and its aridity threshold. Measured plant aboveground biomass (a) and comparisons of aridity threshold values (b) under ambient and nitrogen (N) addition treatments (*n* = 167 sites). Remotely sensed and random forest model-simulated leaf area index (c), and comparisons of aridity threshold values (d) under high (401 ppm) and low (341 ppm) CO_2_ levels. The two comparisons are based on paired datasets of high vs. low N/CO_2_ levels. Data were log-transformed to conform to normality. Box plots in (b) and (d) show the median, upper and lower quartiles, with outlier values represented by black dots. Different letters indicate significant differences in aridity thresholds between high versus low N levels (*P* < 0.001).

**Figure 3.**
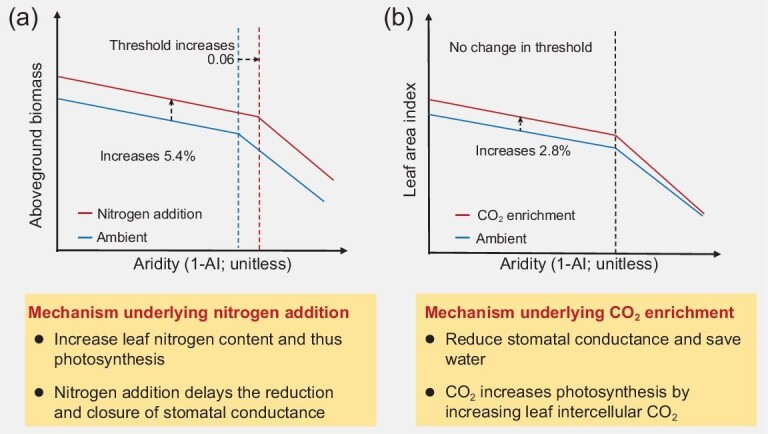
Potential mechanisms underlying the impacts of (a) nitrogen and (b) CO_2_ enrichment on plant aboveground biomass and leaf area index and their aridity threshold. The increase in aboveground biomass (AGB) under nitrogen (N) addition is based on AGB measurements and is expressed as per gram of N added. The increase in leaf area index (LAI) under CO_2_ enrichment is based on LAI simulated by random forest model and is expressed as per 100 ppm of CO_2_ enrichment.

Based on a subset of field experiments (*n* = 167) including AGB measurements under ambient and N addition treatments, we found that N addition significantly delayed the emergence of the aridity-induced threshold by 0.06 aridity units (i.e. from 0.49 to 0.55; *P* < 0.05; Fig. 2a–b). When we accounted for the N addition amount and the types of N compounds (urea, NH_4_NO_3_, NH_4_Cl, etc), the aridity threshold under N addition maintained at 0.55 (Figure S4). When we standardized the biomass measurements by the amount of N added, we observed that AGB increased by ∼5.4% g^−1^ N added (Fig. 3a). Using remote-sensing LAI estimates and model-simulated values from both random forest and TRENDY models, we found that CO_2_ enrichment did not alter the observed aridity threshold (Fig. 2c–d; S5). The observed aridity thresholds of AGB remained consistent when we considered the influence of vegetation type and soil total nitrogen (Figure S6a–b).

### Future changes in the land area crossing the aridity threshold

**Figure 4.**
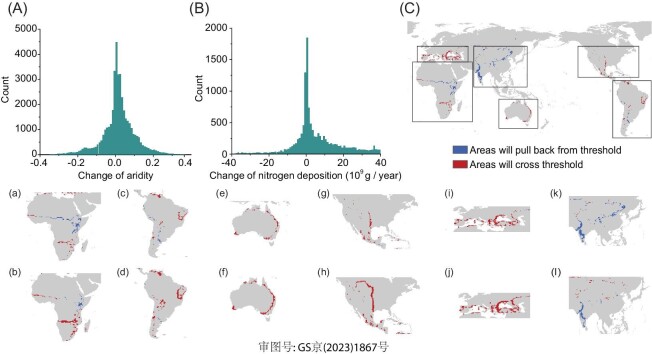
Predicted future changes in land area crossing the aridity threshold for plant aboveground biomass under the RCP 8.5 scenario by 2100. Panels A and B show the frequency distributions of changes in aridity and nitrogen deposition, respectively. Panel C shows predicted future changes in land area crossing the observed aridity threshold; the comparisons between scenarios considering (a, c, e, g, i, k) and not including (b, d, f, h, j, l) the effect of nitrogen deposition are also shown.

To illustrate the effect of N on the aridity thresholds identified for AGB, we estimated future changes in the global land area that will exceed the aridity threshold using the aridity projections under the RCP 8.5 (Fig. 4) and 4.5 (Figure S7) scenarios, respectively. By 2100, and under RCP 8.5 scenario, 63% of land area is projected to experience a change in aridity <0.06 (Fig. 4A). Should the pattern in time reproduce the same way we have seen here across space, we infer that 2.0% of the land area will cross the observed aridity threshold of AGB when the effect of N is included (vs. the 4.0% estimated without considering the N effect; Fig. 4C). These expanding areas are found mostly in Europe, the United States, and Australia. By 2100, 1.7% of the global land surface area will pull back from the observed aridity threshold of AGB when accounting for N effect and aridity reductions, as opposed to 1.1% when the effect of N is not considered. These shrinking areas are located mainly in western India and north-western China (Fig. 4). Overall, we estimate a 0.3% net increase in global land area crossing the aridity threshold if the effect of N and aridity are included, compared to a 2.9% net increase in areas crossing this threshold if the N effect is not accounted for (i.e. only aridity effects are considered; Figure S7). Under the RCP 4.5 scenario, we predict a 0.8% net decrease in land area that will cross the observed aridity threshold when including both N deposition and aridity, compared to a 1.3% net increase if the effect of N is not considered (Figure S7).

**Figure 5.**
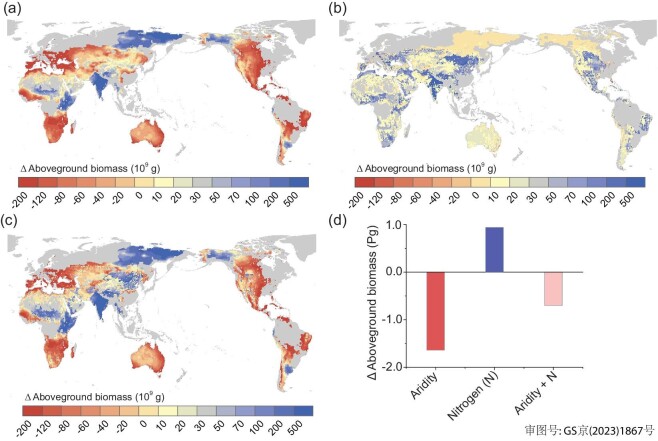
Predicted changes in plant aboveground biomass (AGB). Changes in AGB by 2100 under the RCP 8.5 scenario due to aridity changes (a), nitrogen deposition (b) and the combination of both (c). The overall changes in AGB are summarized in panel d. We include land areas with aridity ≥0, i.e. zones where annual precipitation is ≤ potential evapotranspiration (excluding croplands).

We also estimated the impact of increases in aridity and N deposition on future changes in AGB in areas with aridity values ≥0 (i.e. where annual precipitation is ≤ potential evapotranspiration) under both the RCP 8.5 (Fig. 5a–c) and 4.5 (Figure S8) scenarios. By 2100, and under the RCP 8.5 scenario, aridification will lead to an overall 1.72 Pg decrease in AGB (Fig. 5d). However, when both changes in aridity and N deposition are considered, AGB will decrease by 0.68 Pg.

## DISCUSSION

We detected a sharp decline in measured AGB when crossing the transition point from dry sub-humid to semi-arid climates (aridity = 0.50). This result is broadly consistent with the aridity threshold of 0.52–0.54 observed when using productivity proxies such as the normalized difference vegetation index [4], aboveground biomass carbon and vegetation optical depth (a satellite dataset capturing the aboveground biomass signal) in global drylands (Figure S9). The three possible mechanisms could explain the abrupt decay in AGB observed once this aridity threshold is crossed. First, compared to dry-subhumid systems, the vegetation of semiarid ecosystems is primarily influenced by aridity (i.e. water availability) (Figure S10). For example, Liu *et al.* (2020) [40] found an increasing control of soil moisture on chlorophyll fluorescence beyond an aridity value of 0.50. Second, plants adapt their traits and physiologies to cope with a water-limited environment at the cost of a slower photosynthesis rate [41], and shorter growth periods [42]. Slower photoassimilates accumulation may be unable to compensate for the depletion of stored reserves (i.e. starch and sucrose) due to respiration costs, resulting in less photoassimilate translocation towards the aboveground biomass [43,44]. Finally, to maximize water and nutrient uptake, plants tend to allocate more photoassimilates to belowground roots as both soil water and nutrient availability decreases in a dry environment [45–50]. This is evident from our threshold analysis using >3000 measurements of root:shoot ratios, which increased in an abrupt way when transitioning from dry-subhumid to semi-arid climates (Fig. 1d).

Nitrogen addition delayed the emergence of the observed aridity threshold (Fig. 3). There are three main explanations for the N effect on the aridity threshold. First, higher leaf N would translate into a larger photosynthetic capacity according to the photosynthesis of Fick's law [31]. Leaf N and stomatal conductance represent N and water inputs for photosynthesis, respectively. N addition would increase leaf N. At a given stomatal conductance, higher leaf N translates into a larger drawdown of internal CO_2_ and thus a higher photosynthetic rate. Therefore, plant water use efficiency can be improved under N addition, which contributes to the delay of the emergence of the aridity threshold for plant photosynthesis and biomass. Second, N addition delays the reduction and closure of stomatal conductance [51,52]. The mechanism of delaying the reduction and closure of stomatal conductance under N addition is particularly important in a warming climate, as keeping stomata open can cool plant leaves and protect them from irreversible heat damage [53]. This is increasingly crucial for water limited environments, since current temperatures appear to approach or exceed the optimum for photosynthesis [3,54]. Third, numerous studies have consistently demonstrated the positive effect of N on plant biomass production, encompassing both the aboveground and belowground components [55–57]. This alteration in root allocation, facilitated by N addition, would delay the onset of the aridity threshold by strengthening the water uptake of plants. Therefore, delaying the reduction and closure of stomatal conductance may result in photosynthesis and biomass decays occurring at higher aridity values under N addition.

Leaf area index increased by 2.8% per 100 ppm elevation of CO_2_ (Fig. 3b), which was within the range (0.6%–24.1% increases in AGB per 100 ppm) found in the Free-Air CO_2_ Enrichment experiments [58]. Contrary to our expectation, CO_2_ enrichment did not appear to alleviate the aridity-induced threshold observed. Process models, such as TRENDY, might not be able to simulate the change in aridity threshold due to a lack of underlying mechanisms needed to drive the change. However, this should not have caused the absence of a CO_2_ effect on the aridity threshold we found because our machine learning model, which requires no underlying mechanisms, did not show any CO_2_ effect on the threshold observed. Therefore, our results indicate that CO_2_ enrichment may not mitigate the ecosystem degradation that might occur in drylands once crossing the aridity threshold. While CO_2_ fertilization has been shown to decrease stomatal conductance [17,23,25], this process can lead to reduced transpiration rates. The decreased plant transpiration would reduce leaf cooling and increase both leaf temperature and vapor pressure deficit. As a consequence, excessive stomatal closure may occur, potentially outweighing the benefits of CO_2_ fertilization [59–61]. This may also explain the observed pattern that the CO_2_ effect tends to diminish under high aridity conditions (Fig. 2c).

The effect of N on the emergence of the aridity threshold was particularly noteworthy given our estimates of 0.3% net increase in land area crossing the aridity threshold under a scenario including N deposition, compared to a 2.9% net increase if the N effect is not accounted for. Nitrogen addition increases AGB and, by doing so, increase forage stocks, C sequestration, and the protective effect of vegetation against soil erosion. These are key features to mitigate climate change and desertification impacts in drylands and our results thus suggest that N fertilization may be an ally to evade the negative consequences of aridity thresholds for plant biomass and associated ecosystem services under climate change. However, N addition may lead to local declines in plant biodiversity [62]. Thus, understanding under which environmental conditions such biodiversity declines associated to N addition can occur is an important question that must be explored by future research to guide management actions aiming to maximize the positive effects of N while minimizing the negative ones.

Our findings indicated that N addition may effectively mitigate the negative impact of the projected aridification on plant biomass in water-limited environments. However, there is still ongoing debate about whether greening and productivity would continue to increase under global climate change in recent years [3,52]. Some evidence suggested that the positive trends in greening and productivity might be offset by water stress due to global climate change [63,64]. However, some studies have also found that in highly drought-prone forests, the tree's vulnerability to drought is mitigated by higher N [52]. Moreover, many dryland ecosystems have shown significant greening and enhanced vegetation productivity since the 1980s [3]. Aridification in drylands is a result of potential evapotranspiration increase that exceeds a concurrent increase in precipitation [2,65–67]. Therefore, higher water use efficiency under higher N together with increasing precipitation amount might support a persistent higher plant biomass. Our finding that N addition delays the aridity threshold advances our understanding of vegetation productivity and greenness under global environmental change.

Several limitations in our study should be acknowledged. The space-for-time substitution approach used, while proven highly useful in ecological studies [68], is limited in assessing how and at what speed the aridity threshold can occur through time [69]. Since measurements with sufficiently long timeframes are unavailable, the space-for-time approach may serve as the best substitute if we are focusing on prediction at a decadal time scale (e.g. about 80 years by 2100) [68]. Second, our work is limited by the scarce field sites in drylands in which CO_2_-enrichment studies can be performed, so more field studies will have to be undertaken to redress this gap in our knowledge. To overcome this limitation, we coupled remote sensing LAI and multi-model attributed CO_2_ effects on LAI, which may represent a good alternative if sufficient field measurements are unavailable. The estimation of LAI is based on measuring leaf surface area, and in regions with high vegetation density, leaf overlap can occur, which may potentially result in an underestimation of biomass. However, it is worth noting that our study areas have an aridity index (AI = precipitation/potential evapotranspiration) ≤1, that is, annual precipitation is equal to or less than potential evapotranspiration, resulting in relatively low vegetation cover. In such regions, LAI can still serve as a reliable indicator of plant biomass. Third, our findings help to understand how N and CO_2_ enrichment influence the observed aridity thresholds. However, it is important to note that our results do not provide the specific mechanisms that drive these thresholds. Investigating the underlying mechanisms calls for a different framework and should be the focus of future research endeavors.

## CONCLUSION

Using multiple field and remote sensing datasets, we identified aridity-induced thresholds for plant biomass and how they are affected by N and CO_2_ enrichments. We found that AGB decays sharply and root:shoot ratios abruptly increase once climate shifts from dry sub-humid to semi-arid climates. This finding suggests that careful management is required in areas moving towards a semi-arid climate and, for example, grazing intensities should be significantly reduced to prevent greater decay in plant AGB and cover. We also show that N addition, but not CO_2_ enrichment, delayed the emergence of this aridity threshold. The delayed emergence of the aridity threshold, together with increases in AGB under N addition (around 5% per gram of N), are expected to greatly mitigate the negative effect of aridification on vegetation in water limited ecosystems (precipitation ≤ potential evapotranspiration) worldwide. These findings help disentangle the effects of changes in aridity, as well as of N and CO_2_ enrichment, on plant biomass, which are critical for improving forecasts of vegetation responses to global environment changes.

## METHODS

### Collection of plant biomass measurements

Our study focused on sites with an aridity index (AI = precipitation/potential evapotranspiration) ≤1, i.e. sites where the annual precipitation is ≤ potential evapotranspiration. These areas include all drylands (AI < 0.65) and also some areas with conditions close to drylands whose future conversion into drylands has been forecasted by several studies [2,70]. We preferred to focus on areas with an overall water deficit rather than pure drylands to investigate whether or not the AI value that separates drylands and non-drylands (0.65) is also a threshold for plant biomass, which is of further relevance regarding forecasted dryland expansion [2]. Given that greater AI values mean fewer arid areas, and to facilitate the interpretation of our results, we used 1 − AI as a surrogate of aridity [71].

We compiled plant biomass measurements from published studies, by conducting searches of Web of Science and Google Scholar using the keywords ‘aboveground biomass’ and ‘belowground biomass’. We collated AGB and BGB measurements from 187 published studies (from 1965 to 2018) that met the following criteria: (1) data were obtained from field measurements, (2) studies included coordinate information and (3) study sites had an aridity value ≥0. Most data were collected directly from the main text or supplementary tables of selected papers, although some values were digitally extracted from figures using GetData Graph Digitizer, version 2.22 (http://getdata-graph-digitizer.com/). To minimize the impact of ecosystem restoration on plant biomass/LAI, we implemented a two-fold approach. First, to avoid the potential influence of human activity on plant biomass, we carefully selected our study sites by excluding cropland areas and sites with a Human Footprint Index (HFI) greater than 50% or a percent annual burn area averaging over 30%. Second, in the case of forest ecosystems, we specifically focused on mature or old-growth forests that had stands exceeding 80 years to minimize the influence of afforestation [72]. The Global Human Footprint Dataset (HFI) was obtained from the Wild 220 Project (http://sedac.ciesincolumbia.edu/data/set/wildareas-v2-222human-footprint-geographic). The percent annual burn area was obtained from GFED4 biomass burning emissions dataset (https://daac.ornl.gov/VEGETATION/guides/fireemissions v4.html). The forest age datasets were obtained from the ForestAgeBGI datstet (https://doi.org/10.17871/ForestAgeBGI.2021). For studies including data from multiple years, we averaged values from different years at each site to eliminate the effects of interannual precipitation change and used these mean values in all of our analyses. Overall, we compiled 1353 AGB and 462 BGB measurements under ambient conditions, and 334 AGB measurements from 167 sites where N addition experiments had been conducted (Figure S1). The biomass data were mostly measured during the peak growing season. In some experiments, nitrogen was directly added in the form of granules [73,74], some others were added as an aqueous solution. In the cases of aqueous solution, an equivalent amount of distilled water is usually added to the control plots to account for the effect of increased water [75]. The amount of water addition was equivalent to less than 3 mm of extra precipitation each year [76,77], which was not expected to influence plant biomass. We calculated root:shoot ratios using studies reporting both AGB and BGB (142 measurements). Finally, we also obtained 2951 measurements of root:shoot ratios from a recently published study at a global scale [78].

### Remote sensing leaf area index

The LAI quantifies the amount of foliage in the plant canopy, which is a major driving factor of net primary production, water and nutrient use, and carbon balance [79]. We used NASA’s Global Inventory Modeling and Monitoring Study third-generation dataset of remote sensing LAI (GIMMS LAI 3 g) [80], which was available for the period 1982–2016. We calculated the growing season-integrated LAI for each year and for each 0.5° × 0.5° grid cell of global vegetated areas following the method used by Zhu *et al.* (2016) [27]; however, based on FAO vegetation maps, we classified vegetation types and excluded areas of agriculture, urban landscapes or water bodies [81] to avoid outliers from agricultural and urban lands, since changes in the LAI in these areas are more likely to be driven by human activity (e.g. irrigation) than by changes in aridity.

### The CO_2_ effect on the leaf area index estimated by a machine learning model

Using a similar method to that of Yuan *et al.* (2019) [82], we constructed random forest (RF) machine learning models for simulating LAI driven by climatic factors (air temperature, precipitation, radiation, wind speed, and vapor pressure deficit), atmospheric CO_2_ concentrations, and other background factors (tree age, vegetation type, soil total nitrogen and phosphorus). The background factors reflected the difference in the initial conditions among grid cells, and were not assumed to change during the RF model simulations. We assumed that other potentially unexplained human and natural factors were included in the error term of the RF model. In each vegetation grid cell, we selected 33 years of satellite-observed LAI from the total 34-year period (1982–2015) as training data to develop the RF model, and used the remaining year of LAI as testing data for cross-validation. The RF was run 34 times to ensure that data from all years were selected for model validation. Using the constructed RF model, we predicted the LAI under two factorial simulations: (1) driven by both climate variables and changing CO_2_ over time (1982–2015), and (2) with CO_2_ kept constant at its 1982 value (∼341 ppm) but with climate variables varying over time. The two factorial simulations differed only in CO_2_. The mean values of the 34 simulations were used in our analyses. We compared the aridity thresholds of LAI between the two factorial simulations, which had a 60 ppm difference in CO_2_ (∼401 vs. 341 ppm).

The historical climate fields were obtained from the Climate Research Unit (CRU) and TerraClimate dataset (https://www.climatologylab.org/terraclimate.html), and global atmospheric CO_2_ concentrations were obtained from Greenhouse gases Observing SATellite (GOSAT) data [82]. The soil total nitrogen at 0–30 cm were obtained from the Soil Grid dataset [83], and the total soil phosphorus dataset (0–30 cm) were obtained from the dataset in He *et al.* (2021) [84]. We acquired data on tree age from the forest age dataset [85]. Cross-validation suggested that the RF model simulated LAI matched the satellite observed LAI very well (Figure S11), with correlation coefficients (R^2^) over 0.70 across 96% of vegetated areas worldwide. The root mean square errors obtained were less than 0.10 (1% of the growing season integrated LAI value) in 99.4% of vegetated areas worldwide. These results imply that the RF model used can accurately simulate LAI.

### Assessing the effect of CO_2_ on the leaf area index by using dynamic global vegetation models

We evaluated the effect of CO_2_ on LAI by using the ensemble of 12 Dynamic Global Vegetation Models from the ‘trends and drivers of the regional scale sources and sinks of carbon dioxide’ (TRENDY; see details in Table S1) project [39]. This project performed a factorial set of model simulations over the 1982–2016 period, forced with three factors: CO_2_, climate and land use. We used a multi-model ensemble mean growing-season integrated LAI for the period 1982–2016 under two scenarios: S0 (low CO_2_): LAI-simulated with no forcing changes (time-invariant ‘pre-industrial’ CO_2_, climate and land use); and S1 (high CO_2_): LAI-simulated only with varying CO_2_ (time-invariant ‘pre-industrial’ climate and land use). CO_2_ concentrations were around 285 ppm in S0 and 370 ppm in S1. These two scenarios differed only in CO_2_, thereby allowing us to evaluate the effects of CO_2_ enrichment on LAI (LAI_CO2_). We then removed the CO_2_ effect from the remote sensing LAI (LAI_RS_), i.e. LAI_RS_ − LAI_CO2_ and compared the aridity thresholds between the LAI_RS_ and LAI_RS_ − LAI_CO2_, which have an 85 ppm difference in CO_2_ (370 vs. 285 ppm).

Zhu *et al.* (2016) [27] showed that the TRENDY models, according to an optimal fingerprint detection method, give reliable simulations of the CO_2_ effect on LAI. First, they regressed the remote sensing global average LAI time series against the CO_2_ effects simulated by the TRENDY models. Then, they used a residual consistency test that found consistency between the regression residuals and models simulating LAI in the absence of any CO_2_ forcing.

### Detection of aridity thresholds

We extracted the aridity value for each plant biomass measurement and LAI value. We conducted a multiple regression analysis to examine the relationship between plant biomass, vegetation type, and soil total nitrogen. The residuals from this regression were then used as the response variables for plant biomass. The influence of vegetation type and soil total nitrogen was already accounted for in the random forest analysis. We then used a similar method to Berdugo *et al.* (2020) [4] to detect aridity thresholds. Briefly, we fitted linear and nonlinear (quadratic and general additive models) regressions for the plant biomass/LAI (i.e. residuals) versus aridity data, and selected the model with the lowest Akaike Information Criterion (AIC) value. A threshold only occurs when the nonlinear regression fits the data better than the linear regression. In these cases, we then fitted threshold models by segmented (continuous models), step (discontinuous models) and stegmented (discontinuous models) regressions. Each of these models provides a parameter for describing the value in the predictor (i.e. aridity) that shows the shift in plant biomass (regression slope, intercept or slope + intercept for segmented, step and stegmented regressions, respectively). Stegmented is a combination of step and segmented regressions. Again, we used the lowest AIC value to choose the best fitting among the three threshold models (Table S2). If general additive models where the best model when compared to threshold models, we selected the threshold yielded by segmented regressions to evidence the point of maximum curvature of the regression [4]. The *gam* package was used to fit general additive models [86], while the *MASS* package was used to estimate the AIC value. The *chngpt* package was used to fit segmented, step and stegmented regressions [87]. We implemented all the analysis in R 3.5.3.

To further test whether the identified thresholds significantly affected the slope and/or intercept of the fitted lines, we bootstrapped the linear regressions at both sides of each threshold for each variable. We then extracted the slopes and intercept value of each response variable estimated on both sides of the thresholds and tested significance using a Mann–Whitney U test. Furthermore, we bootstrapped the threshold values of the best-fitting models under ambient and N/CO_2_ enrichment, and compared the differences in the observed thresholds using the *t*-test.

### Prediction of future land area crossing the aridity threshold

We adopted a space-for-time substitution approach to predict changes in global land areas that under RCP 4.5 and 8.5 will probably cross the estimated aridity thresholds by 2100. First, we obtained future aridity projections from the Coupled Model Intercomparison Project Phase 5 [2,66]. Second, we mapped the current and future land area with aridity values (1–Aridity Index) that cross the estimated threshold, and then calculated both expanding and shrinking areas under future climatic conditions. Third, given that N addition might result in a different aridity threshold, we calculated the difference in the thresholds observed between N addition and ambient levels of N (Δthreshold). In locations with rising or falling N deposition in the future, we increased or decreased Δthreshold, respectively. We obtained N deposition data from the datasets of the Representative Concentration Pathways (https://tntcat.iiasa.ac.at/RcpDb/) [88,89]. Both aridity and N deposition datasets had a spatial resolution of 0.5° × 0.5°. Since there were limited biomass measurements of CO_2_ enrichment from field experiments to detect aridity-induced thresholds, we used the aridity threshold estimated using remote sensing and multi-model simulations of LAI. All maps were visualized in ArcGIS 10.5 (ESRI, USA).

We also predicted the impacts of future changes of aridity and N deposition on AGB using a space-for-time substitution approach. First, based on the best regression model between the AGB measurements and aridity values under ambient conditions, we predicted future ΔAGB based on changes in aridity (Δaridity) by 2100 under the RCP 4.5 and 8.5 scenarios.


(1) \begin{eqnarray*}{\mathrm{AGB}} = {\mathrm{a}} + {\mathrm{b}} \times {\mathrm{aridity}}\end{eqnarray*}

(2) \begin{eqnarray*}\Delta {\mathrm{AGB}} = {\mathrm{b}} \times \Delta {\mathrm{aridity}}\end{eqnarray*}

Where a and b are the regression coefficients, which might be different before and after the detected aridity thresholds (see Detection of aridity thresholds section).

Second, given that the 167 N addition experiments use different amounts of N, we standardized the AGB difference between N addition and ambient conditions to ‘per unit N added’, i.e. (AGB_N addition_ − AGB_ambient_)/N_addition_. According to the current and future amounts of atmospheric N deposition, we predicted future ΔAGB caused by changes in N deposition (ΔN_deposition_).


(3) \begin{eqnarray*}
\Delta {\mathrm{AGB}} &\!=\!& ({\mathrm{AG}}{{\mathrm{B}}}_{{\mathrm{N}}\,{\mathrm{addition}}}\! -\! {\mathrm{AG}}{{\mathrm{B}}}_{{\mathrm{ambient}}})/{{\mathrm{N}}}_{{\mathrm{addition}}}\\
&& \times \Delta {{\mathrm{N}}}_{{\mathrm{deposition}}}
\end{eqnarray*}

Finally, we predicted future ΔAGB due to changes in both aridity and N enrichment by 2100 under the RCP 4.5 and 8.5 scenarios.

## DATA AVAILABILITY

Data that support the findings of this study is archived in Figshare, https://doi.org/10.6084/m9.figshare.19738318.

## Supplementary Material

nwad242_Supplemental_File
