# Supplementary material for: Nitrogen addition delays the emergence of an aridity-induced threshold for plant biomass
Source: Natl Sci Rev. 2023 Sep 12;10(11):nwad242. doi: 10.1093/nsr/nwad242 (PMC10600907; doi:10.1093/nsr/nwad242)
Supplement: nwad242_Supplemental_File [file nwad242_supplemental_file.pdf]

Supplementary Information

Contents

Table S1. Details of the 12 TRENDY terrestrial ecosystem models used in this study.

Table S2. Best models obtained for each variable.

Figure S1. Global distribution of the plant biomass dataset used in the study.

Figure S2. Nonlinear responses of belowground biomass to aridity.

Figure S3. Nonlinear response of residuals of plant aboveground biomass to aridity.

Figure S4. Impacts of nitrogen enrichment on plant aboveground biomass and the aridity threshold.

Figure S5. Impacts of CO_2 enrichment on leaf area index and its aridity threshold.

Figure S6. Impacts of nitrogen enrichment on residuals of plant aboveground biomass and its aridity threshold.

Figure S7. Predicted future changes in global land area crossing the aridity threshold for plant aboveground biomass under the RCP 4.5 by 2100.

Figure S8. Predicted future changes in the aboveground biomass under RCP4.5.

Figure S9. Nonlinear responses of aboveground biomass carbon and vegetation optical depth to aridity.

Figure S10. Structural equation model testing the effects of environmental factors on aboveground biomass.

Figure S11. Validation of random forest models for simulating leaf area index (LAI).

Table S1. Details of the 12 TRENDY terrestrial ecosystem models used in this study

Model Name	Model name abbreviation	Spatial resolution	References
Community Atmosphere Biosphere Land Exchange	CABLE	0.5° × 0.5°	1
Community Land Model version 4.5	CLM4.5	0.93° × 1.25°	2
Dynamic Land Ecosystem Model	DLEM	0.5° × 0.5°	3
Integrated Science Assessment Model	ISAM	0.5° × 0.5°	4
The Joint UK Land Environment Simulator	JULES	1.875° × 1.25°	5,6
Lund-Potsdam-Jena General Ecosystem Simulator	LPJ-GUESS	0.5° × 0.5°	7
Lund-Potsdam-Jena Wald Schnee und Landscaft version	LPJ-wsl	0.5° × 0.5°	8
Land Surface Processes and Exchanges model of the University of Bern	LPX-Bern	1° × 1°	9
Organizing Carbon and Hydrology in Dynamic Ecosystems	ORCHIDEE	0.5° × 0.5°	10
ORCHIDEE aMeliorated Interactions between Carbon and Temperature	ORCHIDEE-MICT	1° × 1°	11
Vegetation Global Atmosphere Soil	VEGAS	0.5° × 0.5°	12
Vegetation Integrative Simulator for Trace gases	VISIT	0.5° × 0.5°	13

Table S2. Best models obtained for each variable

Variable name	Type	AIC linear	AIC non-linear	Best AIC
Aboveground biomass	Field	1412.77	1220.59	Stegmented = 1211.10
Belowground biomass	Field	838.18	816.66	Stegmented = 818.42
Root : shoot ratio	Field	5247.75	5168.78	Stegmented = 5167.71
Aboveground biomass	Ambient	136.29	118.49	Piecewise = 117.81
Aboveground biomass	N enrichment	138.81	123.54	Stegmented = 118.46
Leaf area index	Ambient	20368.88	18856.48	Piecewise = 18865.5
Leaf area index	CO2 enrichment	19921.58	18286.52	Piecewise = 18295.44

Variables used with their corresponding Akaike (AIC) values after fitting linear, nonlinear and best threshold models. Lower AIC/BIC values indicate a better fit of the model.

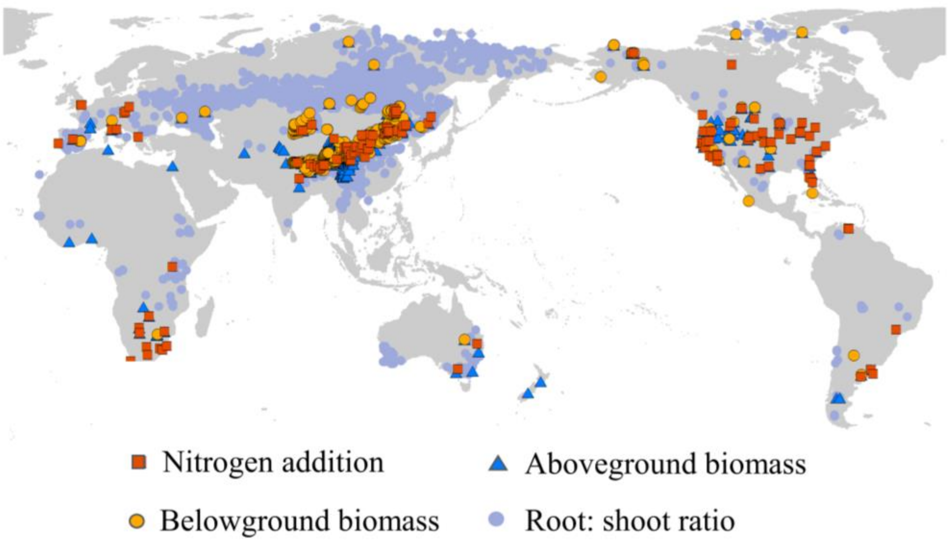

Figure. S1 Global distribution of the plant biomass dataset used in the study.

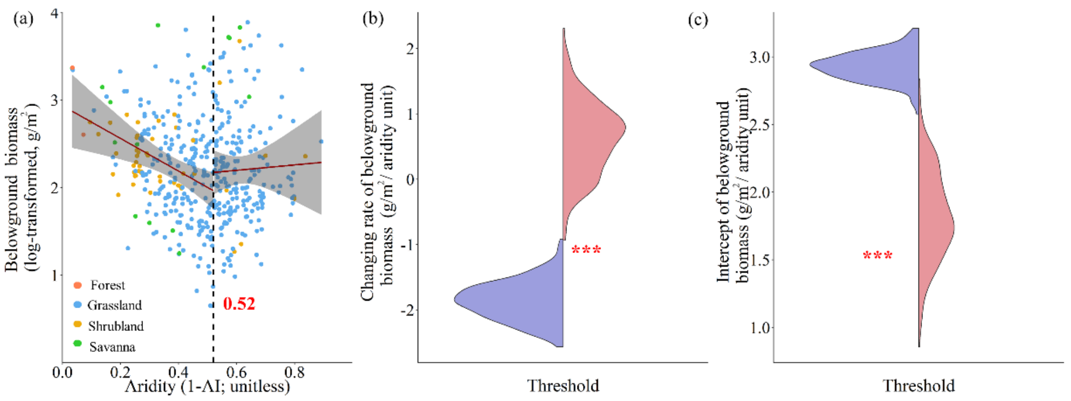

Figure S2. Nonlinear responses of belowground biomass to aridity. Aridity thresholds for measured (a) belowground biomass (n = 462). Solid red lines represent the linear fits on both sides of each threshold. The red numbers and vertical dashed lines represent the identified aridity thresholds. Belowground biomass data were log-transformed to conform to normality. The violin diagrams in panel b show bootstrapped slopes of the predicted fitted trend at the threshold of the two regressions existing at each side of the threshold (purple before the threshold, red after the threshold). The violin diagrams in panel c show bootstrapped intercepts.

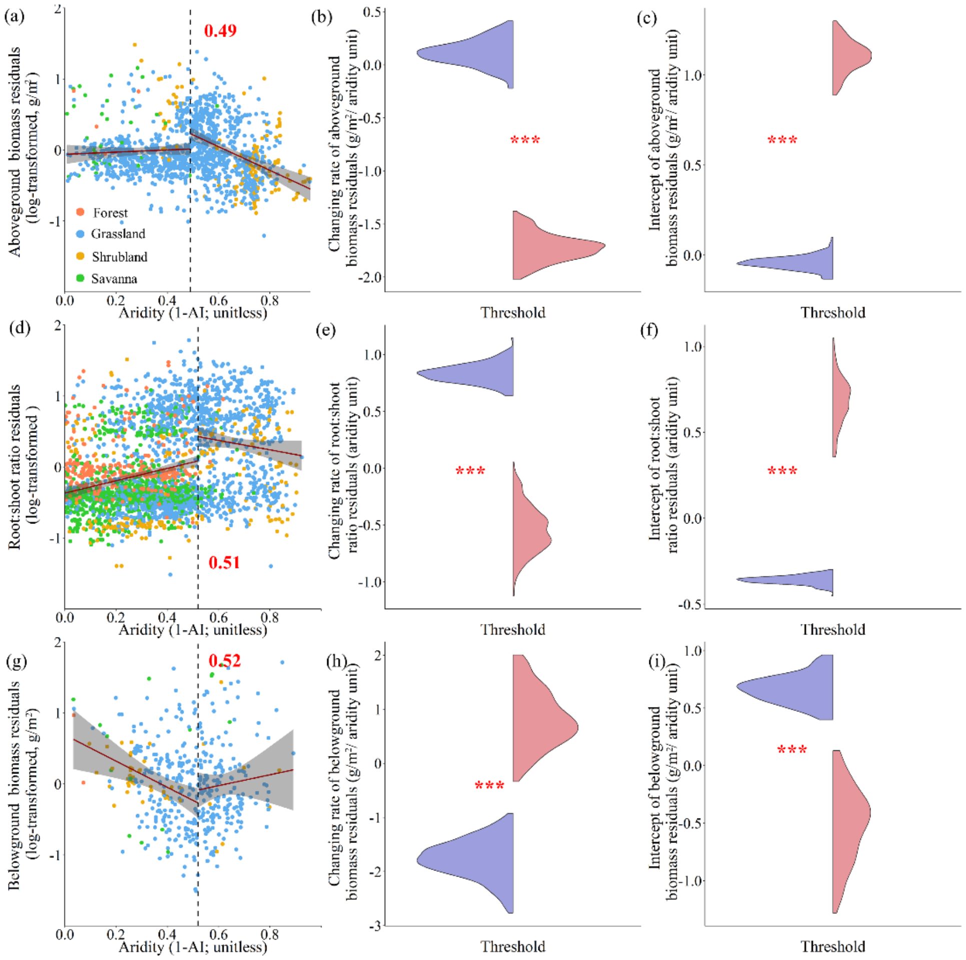

Figure S3. Nonlinear response of residuals of plant aboveground biomass to aridity. Response variables (i.e., partial residuals of plant biomass) were log-transformed to conform to normality. Aridity thresholds for measured (a) aboveground biomass (n = 1353), (d) root-shoot ratio (n = 3093), and (g) belowground biomass (n = 462). Solid red lines represent the linear fits on both sides of each threshold. The red numbers and vertical dashed lines represent the identified aridity thresholds. The violin diagrams in panels b, e and h show bootstrapped slopes of the predicted fitted trend at the threshold of the two regressions existing at each side of the threshold (purple before the threshold, red after the threshold). The violin diagrams in panels c, f and i show bootstrapped intercepts.

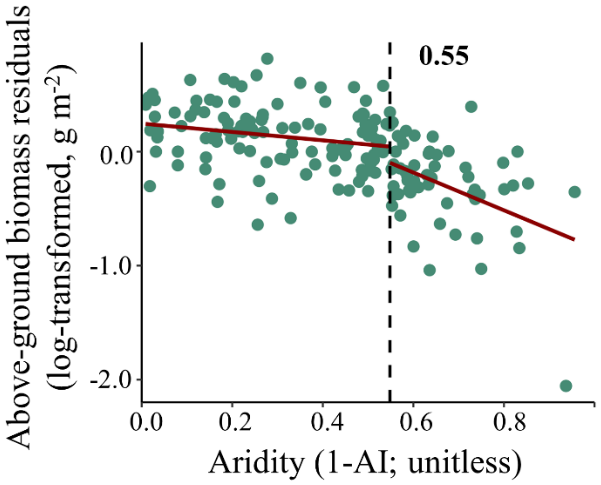

Figure S4. Impacts of nitrogen enrichment on residuals of plant aboveground biomass and the aridity threshold observed. Data were log-transformed to conform to normality.

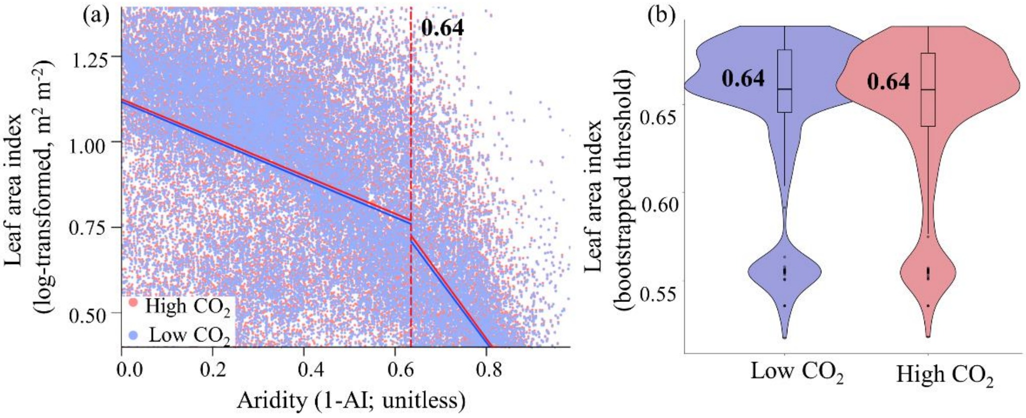

Figure S5. Impacts of CO_2 enrichment on leaf area index and its aridity threshold. Remotely sensed and TRENDY model-simulated leaf area index (a), and comparisons of aridity threshold values (b) under high (370 ppm) and low (285 ppm) CO_2 levels. The comparisons is based on paired datasets of high vs. low CO_2 levels. Data were log-transformed to conform to normality. Box plots in (b) show the median, upper and lower quartiles, with outlier values represented by black dots.

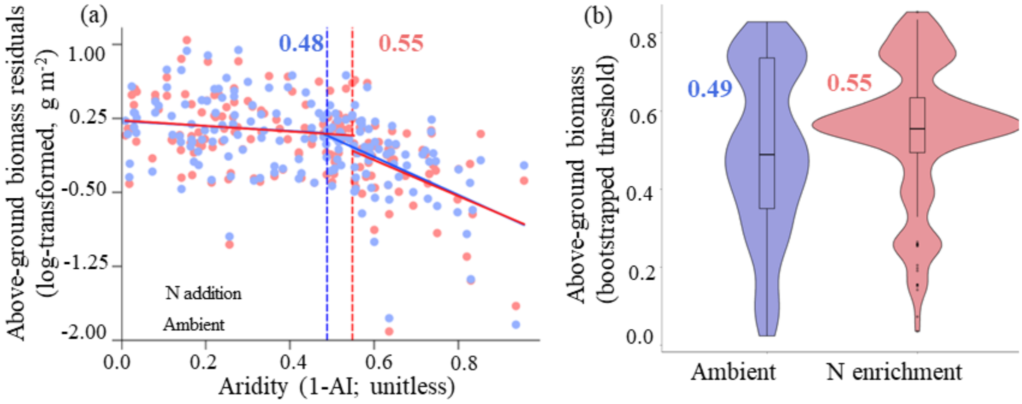

Figure S6. Impacts of nitrogen enrichment on residuals of plant aboveground biomass and its aridity threshold. Response variables (i.e., partial residuals of plant biomass) were log-transformed to conform to normality. Measured plant aboveground biomass (a) and comparisons of aridity threshold values (b) under ambient and nitrogen (N) enrichment treatments (n = 167 sites). The two comparisons are based on paired datasets of high vs. low N levels. Box plots in (b) show the median, upper and lower quartiles, with outlier values represented by black dots. Different letters indicate significant differences in aridity thresholds between high versus low N levels (p < 0.001).

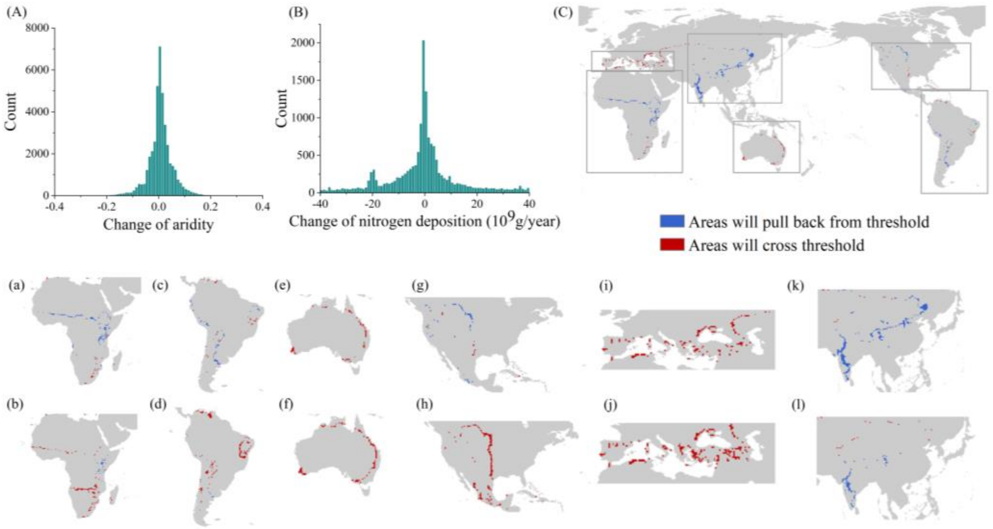

Figure S7. Predicted future changes in global land area crossing the observed aridity threshold for plant aboveground biomass under the RCP 4.5 by 2100. Panels A and B show the frequency distributions of changes in aridity and nitrogen deposition, respectively. Panel C shows the predicted future changes in land area crossing the observed aridity threshold. The comparisons between scenarios considering (panels a, c, e, g, i, k) and without considering (panels b, d, f, h, j, l) the effect of nitrogen deposition are shown.

By 2100, 1.9% of the land area will cross the aridity threshold of AGB when the effect of N deposition is included, versus 2.0% without considering the N effect. These expanding areas distribute mostly in Europe, the United States, and Australia. By 2100, 1.7% of land area will pull back from the aridity threshold of AGB if N deposition is accounted for, whereas only 0.7% will retreat if the effect of N is not considered. These shrinking areas are located mainly in western India and north-western China (Figure S5). Overall, we estimate that there will be a 0.2% net increase in land surface area crossing the aridity threshold under a high-emissions scenario including N enrichment,

compared to a 1.3% net increase in areas crossing the aridity threshold if the effect of N is not considered.

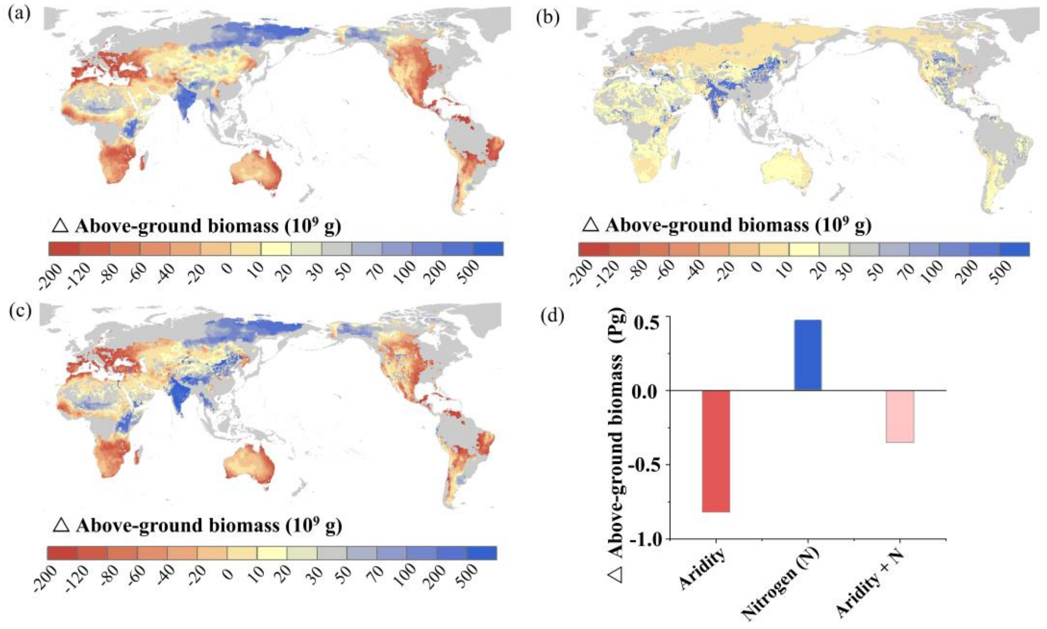

Figure S8. Predicted future changes in the aboveground biomass under RCP4.5. Changes of aboveground biomass (AGB) in 2100 under RCP4.5 due to aridity change (a), nitrogen deposition (b), and the combination of both (c). The overall changes in AGB are summarized in panel d. We include land areas with aridity values ≥ 0, i.e. areas where annual precipitation is ≤ potential evapotranspiration (excluding croplands).

By 2100, and under the RCP 4.5 scenario, aridification leads to an overall 0.8 Pg decrease in AGB. When both changes in aridity and N deposition are considered, AGB will decrease by 0.3 Pg.

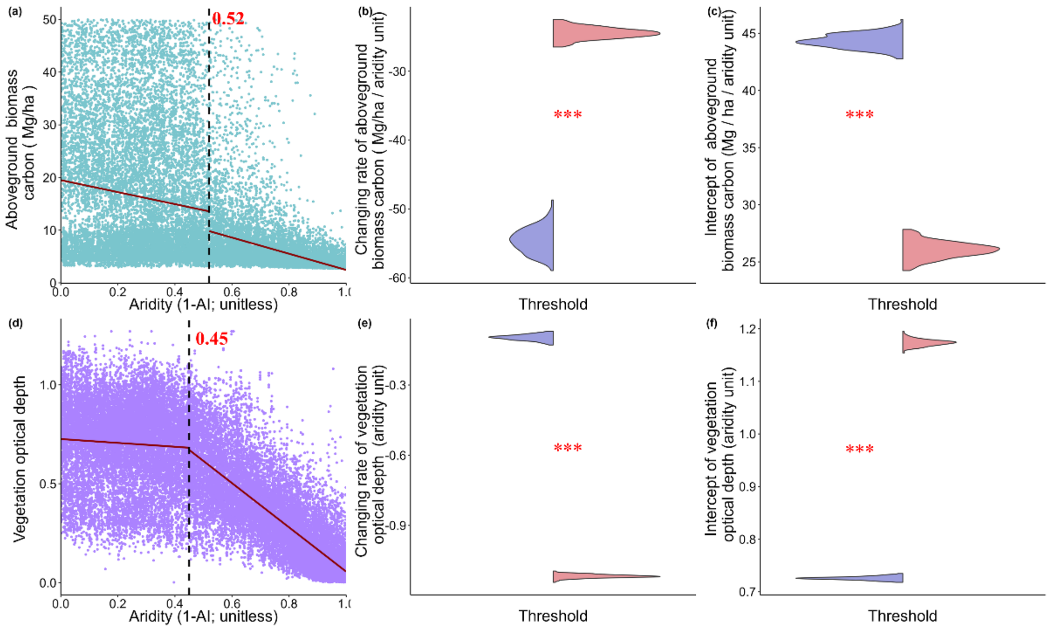

Figure S9. Nonlinear responses of aboveground biomass carbon and vegetation optical depth to aridity. Aridity thresholds for aboveground biomass carbon (a) and vegetation optical depth (d). Solid red lines represent the linear fits on both sides of each threshold. The red insertion numbers and vertical dashed lines describe the identified aridity thresholds. In panels b and e, the violin diagrams show bootstrapped slopes of the predicted fitted trends at the threshold of the two regressions existing at each side of the threshold (purple before the threshold, red after the threshold). In panels c and f, the violin diagrams show bootstrapped intercepts. The asterisk represents a difference before and after the threshold at $p < 0.001$. We obtained remotely sensed global mean aboveground biomass carbon (~ 45% of aboveground biomass) and vegetation optical depth (capturing the aboveground biomass signal) for 1993-2012 at a spatial resolution of 0.5° × 0.5° from Liu et al.[14].

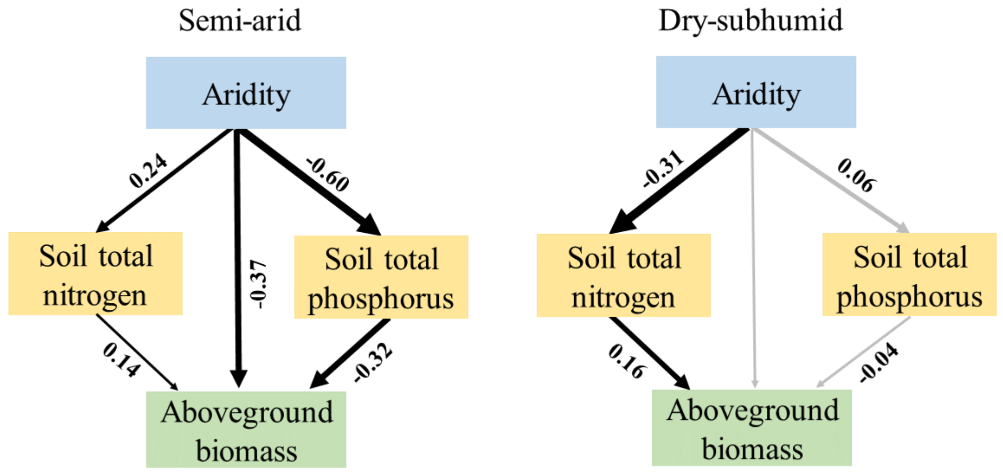

χ^2= 0.17, *df* = 1, *p* = 0.68,
RMSEA=0 , *CFI* = 1.0

$\chi 2$= 3.7, *df* = 1, *p* = 0.06,
RMSEA= 0.06 , *CFI*= 0.97

Figure S10. Structural equation model testing the effects of environmental factors on aboveground biomass. The path coefficients, which indicate the strength of the relationships among the variables, are displayed next to the arrows. The gray path represents a non-significant relationship.

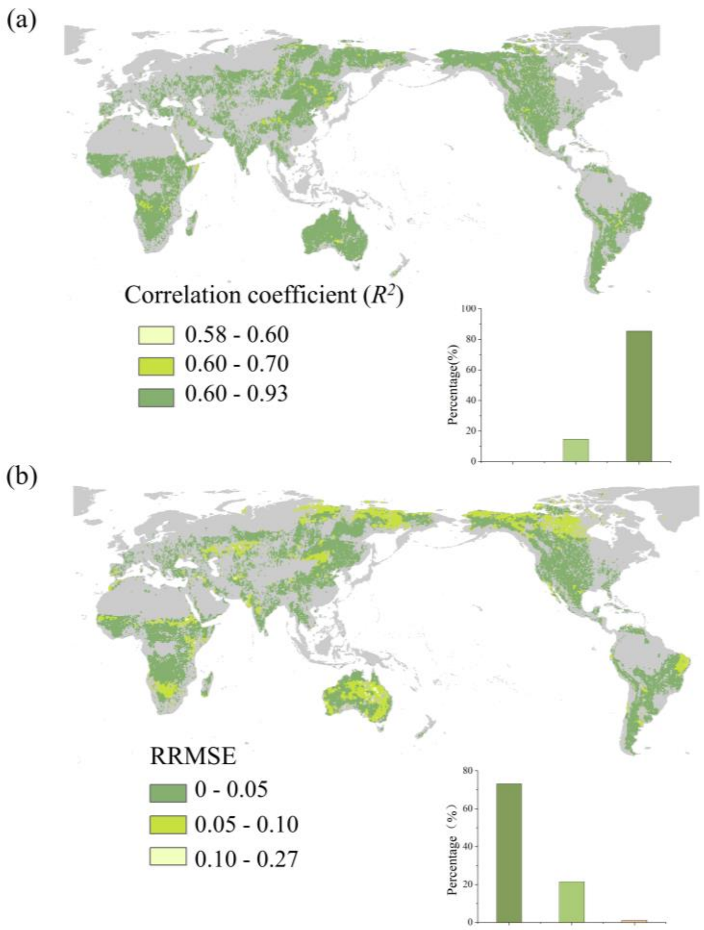

Figure S11. Validation of random forest models for simulating leaf area index (LAI). (a) Correlation coefficient (R^2) between simulated and remote sensing observed LAI, (b) relative root mean square error (RRMSE = RMSE / O, RMSE= $\frac{\sqrt{\sum_{i=1}^{n}(S\text{-}O)^2}}{n}$; S and O indicate the simulated LAI and remote sensing observed LAI, respectively).

References

[1] Wang, Y. P., Law, R. M., & Pak, B. (2009). A global model of carbon, nitrogen and phosphorus cycles for the terrestrial biosphere. *Biogeosciences*, 7(7), 9891-9944.

[2] Oleson, K. W., Lawrence, D. M., Gordon, B., Flanner, M. G., & Zeng, X. (2010). Technical description of version 4.0 of the community land model (CLM). (No. NCAR/TN-478+STR). *University Corporation for Atmospheric Research*.

[3] Tian, H. Q., Chen, G. S., Zhang, C., Liu, M. L., Sun, G., & Vance, E. (2012). Century-scale responses of ecosystem carbon storage and flux to multiple environmental changes in the Southern United States. *Ecosystems*, 15, 674-694.

[4] Jain, A. K., & Yang, X. (2005). Modeling the effects of two different land cover change data sets on the carbon stocks of plants and soils in concert with co2 and climate change. *Global Biogeochemical Cycles*, 19(2), GB2015.

[5] Best, M. J., Pryor, M., Clark, D. B., Rooney, G. G., & Gedney, N. (2011). The joint UK land environment simulator (JULES), model description – part 1: energy and water fluxes. *Geoscientific Model Development*, 4, 677-699.

[6] Clark, D. B., Mercado, L. M., Sitch, S., Jones, C. D., Gedney, N., & Best, M. J. (2011). The joint UK land environment simulator (JULES), model description – part 2: carbon fluxes and vegetation dynamics. *Geoscientific Model Development*, 4(3), 701-722.

[7] Smith, B., & Sykes, P. M. T. (2001). Representation of vegetation dynamics in the modelling of terrestrial ecosystems: comparing two contrasting approaches within European climate space. *Global Ecology & Biogeography*, 10(6), 621-637.

[8] Stich, S., Smith, B., Prentice, I. C., Arneth, A., Bondeau, A., & Venevsky, S. (2003). Evaluation of ecosystem dynamics, plant geography and terrestrial carbon cycling in the LPJ dynamic global vegetation model. *Global Change Biology*, 9(2), 161-185.

[9] Stocker, B. D., Roth, R., Joos, F., Spahni, R., Steinacher, M., & Zaehle, S. (2013). Multiple greenhouse-gas feedbacks from the land biosphere under future climate change scenarios. *Nature Climate Change*, 3(7), 2747.

[10] Krinner, G (2005). A dynamic global vegetation model for studies of the coupled atmosphere-biosphere system. *Global Biogeochemical Cycles*, 19, 1-33.

[11] Guimberteau, M., Zhu, D., Maignan, F., Huang, Y., Yue, C., & Ciais, P. (2018). a land surface model for the high latitudes: model description and validation, *Geoscientific Model Development*, 11, 121–163,

[12] Zeng, N., Mariotti, A., & Wetzel, P. (2005). Terrestrial mechanisms of interannual CO_2 variability. *Global Biogeochemical Cycles*, 19, 1-15.

[13] Kato, E., Kinoshita, T., Ito, A., Kawamiya, M., & Yamagata, Y. (2013). Evaluation of spatially explicit emission scenario of land-use change and biomass burning using a process-based biogeochemical model. *Journal of Land Use Science*, 8(1), 104-122.

[14] Liu, Y.Y., van Dijk, A. I. J. M., de Jeu, R.A.M., Canadell, J.G., McCabe, M.F., Evans, J.P., & Wang, G. (2015). Recent reversal in loss of global terrestrial biomass. *Nature Climate Change*, 5(5), 470–474
